# Molecular insights into human taste perception and umami tastants: A review

**DOI:** 10.1111/1750-3841.16101

**Published:** 2022-03-17

**Authors:** Johan Diepeveen, Tanja C. W. Moerdijk‐Poortvliet, Feike R. van der Leij

**Affiliations:** ^1^ Research Group Marine Biobased Specialties Chemistry Department, HZ University of Applied Sciences Vlissingen The Netherlands; ^2^ Research and Innovation Centre Agri, Food & Life Sciences (RIC‐AFL) Inholland University of Applied Sciences Delft The Netherlands

**Keywords:** flavor, protein, TAS1R1/TAS1R3, taste receptor, umami

## Abstract

Understanding taste is key for optimizing the palatability of seaweeds and other non‐animal‐based foods rich in protein. The lingual papillae in the mouth hold taste buds with taste receptors for the five gustatory taste qualities. Each taste bud contains three distinct cell types, of which Type II cells carry various G protein‐coupled receptors that can detect sweet, bitter, or umami tastants, while type III cells detect sour, and likely salty stimuli. Upon ligand binding, receptor‐linked intracellular heterotrimeric G proteins initiate a cascade of downstream events which activate the afferent nerve fibers for taste perception in the brain. The taste of amino acids depends on the hydrophobicity, size, charge, isoelectric point, chirality of the alpha carbon, and the functional groups on their side chains. The principal umami ingredient monosodium l‐glutamate, broadly known as MSG, loses umami taste upon acetylation, esterification, or methylation, but is able to form flat configurations that bind well to the umami taste receptor. Ribonucleotides such as guanosine monophosphate and inosine monophosphate strongly enhance umami taste when l‐glutamate is present. Ribonucleotides bind to the outer section of the venus flytrap domain of the receptor dimer and stabilize the closed conformation. Concentrations of glutamate, aspartate, arginate, and other compounds in food products may enhance saltiness and overall flavor. Umami ingredients may help to reduce the consumption of salts and fats in the general population and increase food consumption in the elderly.

## 1 INTRODUCTION

The rapidly growing world population and the unsustainable meat industry are major driving forces for the exploration of alternative, more sustainable, sources of protein. Firstly, the protein demand is predicted to increase drastically by 2050 due to the growing world population and increasing number of middle‐income families (Henchion et al., 2017). Secondly, the vast increase in meat production has put tremendous strain on the global ecosystem due to its environmental impacts (Willet et al., 2019). Promising alternative protein sources like seaweed and other non‐animal‐based foods need to be appreciated by a bigger audience to make an impact. Seaweeds are rich in protein, healthy lipids, minerals, and vitamins and most seaweeds are naturally high in l‐glutamate, which elicits umami taste. Analytical methods are available to determine the key contributors to umami and to predict taste intensity and active values (Moerdijk‐Poortvliet et al., 2022). A good taste is key in persuading the general public to accept novel food products and to start including it into their diets. Understanding the mechanisms behind tasting in general and umami tasting in particular may further the palatability of seaweed‐containing food products. This paper aims to evaluate the current state of research on the five gustatory tastes, with special focus on umami tastants.

## 2 TASTE BIOLOGY

### 2.1 Mammalian taste

The human sensory system mediates the perception of the environment, including chemical (taste and olfaction) and physical (e.g., vision, temperature, and sound) aspects responsible for identifying harmful substances and regulating nutrient intake. There are five recognized taste qualities: Sweet, bitter, sour, salty, and umami. Sensations such as pungency (spiciness) and astringency are largely detected by the somatosensory system and are thus not included in the five gustatory taste qualities. Each taste quality conveys nutritional or physiological necessities or potential dietary risks. Sweet‐tasting foods signal the presence of carbohydrates, a major energy source, and umami signals the presence of some amino acids (Chaudhari et al., 2009) critical for vital processes such as the building of hormones, neurotransmitters, and muscle mass (Wu, 2013). Salty‐tasting foods govern the intake of minerals, essential for nerve and muscle function, and in regulating body fluids. Sourness signals the presence of dietary acids and low pHs are generally regarded as unpleasant. Spoiled foods are generally acidic and are consequently avoided. Lastly, humans and other mammals show an innate aversion to highly bitter‐tasting foods, which is postulated to be a defense mechanism against poisonous substances (Wilson et al., 2019).

### 2.2 Gustatory system

**FIGURE 1:**
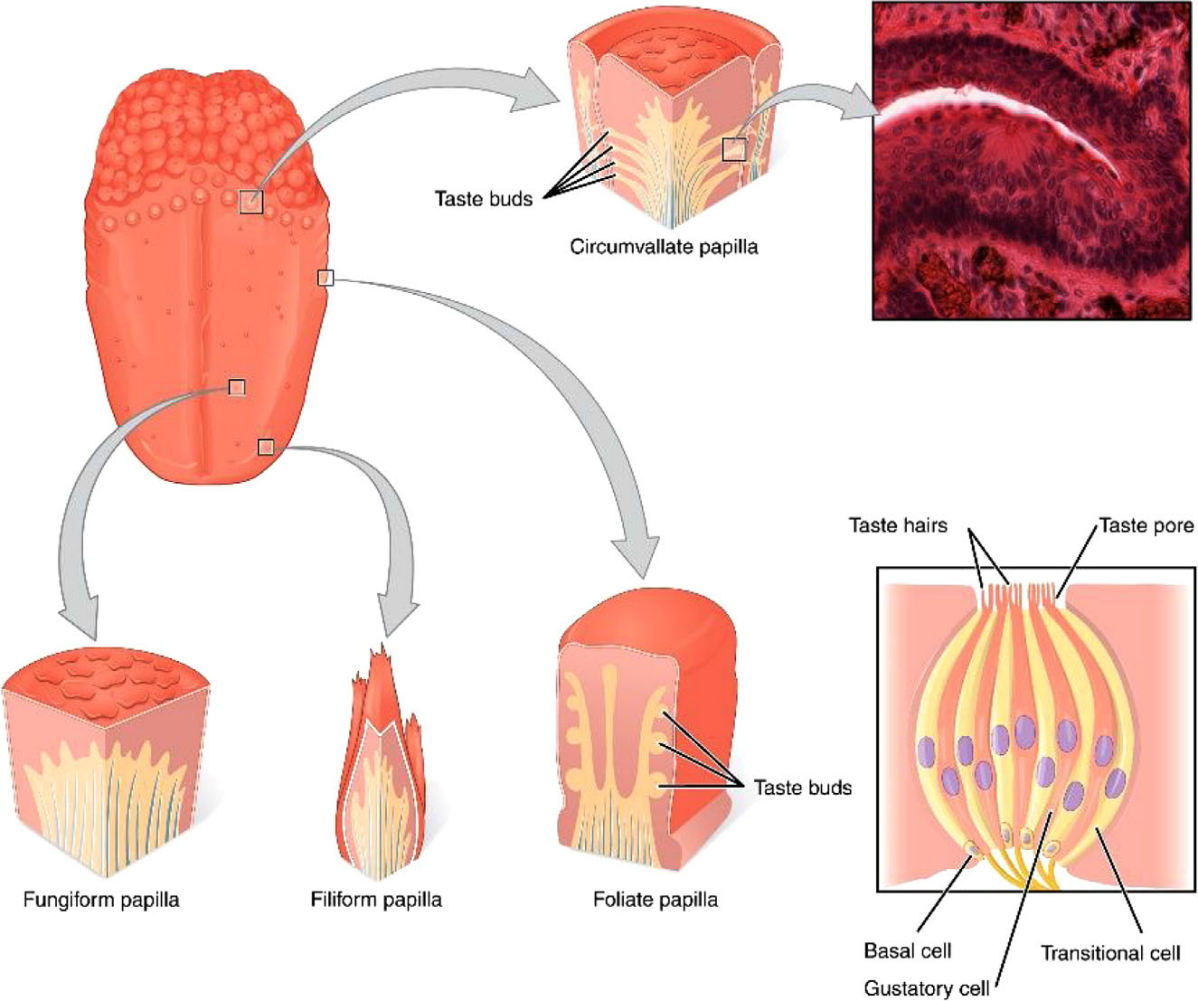
Anatomy of the human tongue. On the surface of the tongue (upper left) four types of lingual papillae (circumvallate, fungiform, filiform, and foliate) can be distinguished as indicated. A cross‐section of a circumvallate papilla is shown upper right. A schematic representation of a taste bud with basal, gustatory, and transitional cells is shown lower right. Taste hairs of the gustatory cells are accessible to saliva via taste pores (OpenStax Anatomy & Physiology, 2016)

The first structures of the gustatory system, the lingual papillae, give the tongue its distinctive rough surface. The lingual papillae include circumvallate‐, fungiform‐, and foliate papillae, each of which hold taste buds that in turn contain the taste receptor cells that are responsible for transducing the taste signal (Figure 1). The lingual papillae include filiform papillae that perceive touch, temperature, and pain but do not contain taste buds and cannot perceive taste (Gravina et al., 2013).

The hairlike structures (microvilli or taste hairs) at the taste bud opening (taste pore) mediate the binding of the tastants dissolved in the saliva to the taste receptor of a gustatory cell. These microvilli also increase the surface area and the absorption efficiency of the cell.

A taste bud contains three distinct cell types (type I–III). Type I cells account for 50% of all taste bud cells, while type II cells make up 20–30% and type III cells about 10% (Miura et al., 2001). Type I cells enclose other cell types and regulate (re)uptake and degradation of neurotransmitters (van der Werf et al., 2017), predominantly adenosine triphosphate (Finger et al., 2005). Type II cells are taste receptors containing gustatory cells that detect and transduce sweet, bitter, and umami stimuli (Zhang et al., 2003). Each taste is detected by different receptors, yet downstream signal transduction cascades remain largely identical among different type II receptors (van der Werf et al., 2017). Type III cells contain receptors which detect sour and, likely, salty stimuli (Wilson et al., 2022).

### 2.3 Cell‐surface receptors

Taste compounds are detected by protein receptors, more specifically G protein‐coupled receptors (GPCRs). GPCRs are the largest and most diverse group of membrane receptors in eukaryotes. Humans have nearly a thousand GPCRs, all of which are important in various signaling pathways. These pathways regulate a variety of processes in cognition (Thathiah & De Strooper, 2011), metabolism (Carpino & Goodwin, 2010) including endocrinological disorders (Vasseur et al., 2003), inflammation (Vroon et al., 2006), and immunity (Dragun et al., 2009). It is estimated that about 30–50% of the available drugs target for GPCRs (Cheng et al., 2012).

**FIGURE 2:**
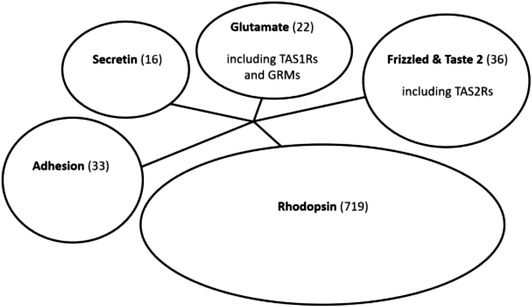
Phylogenetic relationship of the five classes of G Protein‐Coupled Receptors (GPCRs). Based on an alignment of 826 human GPCRs, Lv et al. (2016) produced a phylogenetic tree that is shown in a compiled way. The number of members within each class is indicated between brackets. The taste receptor families TAS1R and GRM belong to the glutamate class whereas the TAS2R family belongs to the Frizzled & Taste 2 class

GPCRs can detect (i.e., bind with) structurally different signaling molecules such as odorants, neurotransmitters, and hormones (Pierce et al., 2002). Light‐sensitive GPCRs such as rhodopsin are involved in visual phototransduction (Lamb, 2013). GPCRs can be classified into five classes and further divided into subfamilies based on sequence similarities (Lv et al., 2016). The GPCRs capable of detecting sweet and umami taste belong to class of glutamate GPCRs (Figure 2).

### 2.4 Structure of GPCRs and G proteins

**FIGURE 3:**
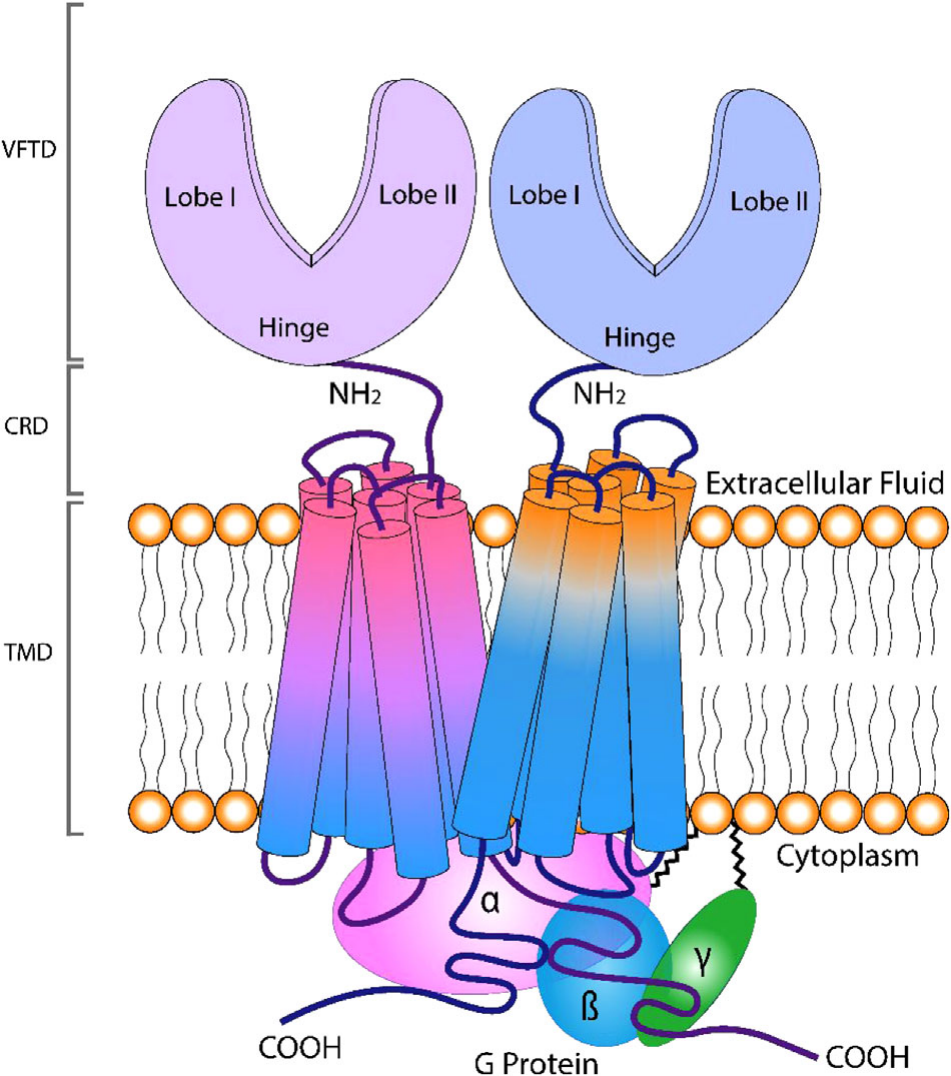
The umami taste receptor dimer TAS1R1+TAS1R3 in the plasma membrane and the intracellular G‐protein heterotrimer. The taste receptor heterodimer consists of TAS1R1 and TAS1R3, each with seven transmembrane protein segments (trans membrane domain, TMD) that anchor them in the taste cell membrane. Each monomer has two lobes on the extracellular side, together known as the Venus flytrap domain (VFTD). These N‐terminal lobes are attached to the TMD via a cysteine‐rich domain (CRD). The lobes, TMD, and CRD all function as possible ligand binding sites. Black jagged lines represent membrane attachment of the G proteins α and γ that reside with G protein β on the cytoplasmic side. The G protein trimer interacts with the C‐termini of the receptor proteins

GPCRs are embedded in the plasma membrane and share, despite their versatility, a common structure of seven transmembrane helices, which loop both intra‐ and extracellularly (Figure 3). The extracellular loops act as ligand‐binding site. Upon activation of a GPCR, linked intracellular heterotrimeric G proteins initiate a cascade of downstream second messenger pathways that activate the afferent nerve fibers (Gravina et al., 2013). The extracellular GPCR loops contain cysteine residues (known as the cysteine‐rich domain, CRD), that form disulfide bonds which stabilizes the GPCR structure. The heterotrimeric G protein that associates with GPCRs consists of monomer Gα and heterodimer Gβγ. Subunits Gα and Gγ are attached to the plasma membrane via lipid anchors provided by palmitoylation (Figure 3).

## 3 TASTE RECEPTION

### 3.1 Umami taste

First discovered and coined by Ikeda (1909), umami, Japanese for “deliciousness” is the distinct savory taste of broths, but also of cooked meat, (shell)fish, tomatoes, mushrooms, and certain cheeses (Kurihara, 2015; Zhang et al., 2013). The first compound to be identified reported to elicit umami was glutamic acid (Ault, 2004; Ikeda, 1909). Since then, many more compounds have been found to elicit or enhance umami. Various terms have been used to describe the umami taste, mainly focused on the flavor characteristics of continuity, mouthfullness, impact, mildness, and thickness. Umami has been described as a “feeling of satisfaction” and has been perceived as an aromatic, despite being mostly odorless (Yamaguchi & Ninomiya, 1998).

### 3.2 Umami taste receptors

Umami taste is mediated by two types of taste receptors. The glutamate class (Figures 2 and 3) GPCR dimer TAS1R1+TAS1R3 functions as an l‐amino acid receptor (Nelson et al., 2002) and is considered to be the predominant umami taste receptor. The contribution of the TAS1R1 monomer is essential in mediating umami taste (Mouritsen & Khandelia, 2012).

The second type of umami taste receptors, also of the glutamate class (Figure 2), are four taste‐specific metabotropic glutamate receptors, GRM1 to GRM4, previously called mGLUR1 to 4 (Chaudhari et al., 2009; Yasumatsu et al., 2015). These GRMs are activated by glutamate and analogs such as l‐(+)−2‐amino‐4‐phosphonobutyrate (Pal Choudhuri et al., 2016; Yasumatsu et al., 2015). In the central nervous system, especially in the brain, glutamate is the main excitatory neurotransmitter and also the precursor of the main inhibitory neurotransmitter, γ‐aminobutyric acid (GABA; Petroff, 2002). The GRMs that mediate umami tasting are short versions of the glutamate neuroreceptors, missing large parts of the extracellular N‐terminal domain (Kurihara, 2015; San Gabriel et al., 2005). Both GRM1 and GRM4 are expressed in taste buds but are about one hundred times less sensitive to glutamate than the brain‐variants (Kurihara, 2015; San Gabriel et al., 2005).

### 3.3 Sweet taste receptors

Also belonging to the TAS1R family, the sweet receptor TAS1R2+TAS1R3 functions as a broadly tuned sweet sensor (Nelson et al., 2001). In contrast to the l‐amino acid receptor TAS1R1+TAS1R3, the sweet receptor TAS1R2+TAS1R3 senses, next to sweet amino acids such as glycine and d‐tryptophan, also the sweet amino acid dimer aspartame and various sweet proteins such as brazzein, monellin, and thaumatin (Temussi, 2002). The large cavity in the extracellular domain of the TAS1R3 receptor and the molecular structure of sweet protein are key factors for sensing sweet protein (Temussi, 2011).

### 3.4 The common component TAS1R3

TAS1R3 is the common component of both the sweet and umami taste receptor (Zhao et al., 2003). TAS1R3 only fulfills its role in the detection and transduction of either sweet or umami in the presence of its respective partner, TAS1R2 or TAS1R1.

TAS1R2‐positive cells invariably express TAS1R3 (Nelson et al., 2001). However, cells that express TAS1R3 “alone” are formed in substantial amounts on the tongue and palate epithelium (roof of mouth). Behavioral and physiological evidence exists for TAS1R3 functioning as homodimer (Zhao et al., 2003). However, as a homodimer, TAS1R3 functions as a low‐affinity sweet taste receptor (Table 1). It responds moderately to high concentrations (>300 mM) of natural sugars and, contrary to TAS1R2+TAS1R3, does not respond to d‐amino acids or sugar substitutes (Zhao et al., 2003). Low caloric sweeteners cannot reach the same intensity of sweetness compared to natural sugars at higher concentrations, which may be explained by the partitioned expression of sweet taste receptor cells (Zhao et al., 2003).

**TABLE 1:** Sugar response of homodimer TAS1R3 and sweet heterodimer TAS1R2+TAS1R3 measured by increase of [Ca^2+^]i (Zhao et al., 2003)

**Taste receptor/sweet substance**	**Sucrose (300 mM)**	**Sucrose (500 mM)**	**Saccharin (300 mM)**
TAS1R2+TAS1R3 (heterodimer)	++	+++	+
TAS1R3+TAS1R3 (homodimer)	–	+	–

Receptor heterodimer TAS1R2+TAS1R3 responds to sweet substances of varying concentrations. Homodimer TAS1R3 responds to high but still physiologically relevant concentrations of natural sugars only. No homodimer TAS1R3 response was observed for the sugar substitute saccharin.

### 3.5 Bitter taste receptors

Whereas umami and sweet are governed by TAS1 receptor dimers, the bitter taste is mediated through TAS2 receptors that may act either as monomers or dimers, although there is evidence for their functionality as oligomers (Kuhn et al., 2010). In humans and mice, the TAS2R family consists of 25 taste‐specific GPCRs linked to bitter taste (Wooding et al., 2021). Unlike the sweet and umami GPCRs, the TAS2R family is closely related to the so‐called frizzled GPCRs (Lv et al., 2016; Figure 2), receptors that are important for body development in multicellular animals. Structure–function studies to understand ligand binding have been conducted on 9 of the 25 TAS2Rs, including TAS2R4 (Singh et al., 2011), TAS2R10 (Born et al., 2013), TAS2R14 (Levit et al., 2014), TAS2R16 (Sakurai et al., 2010), TAS2R30 (Pronin et al., 2004), TAS2R38, TAS2R43, TAS2R44, and TAS2R46 (Brockhoff et al., 2010). See Table 2 for a list of the identified bitter taste receptors. A large repertoire of TAS2R subsets are expressed in a single taste receptor cell (Wooding et al., 2021), which suggests that these receptors together function as a broadly tuned bitter sensor.

**TABLE 2:** List of identified human TAS2 receptors and the chromosomal positions of the genes that encode them (HUGO Gene Nomenclature Committee, 2021; www.ncbi.org)

**Taste receptor symbol**	**Chromosome**
TAS2R1	5p15.31
TAS2R3	7q34
TAS2R4	7q34
TAS2R5	7q34
TAS2R7	12p13.2
TAS2R8	12p13.2
TAS2R9	12p13.2
TAS2R10	12p13.2
TAS2R13	12p13.2
TAS2R14	12p13.2
TAS2R16	7q31.32
TAS2R19	12p13.2
TAS2R20	12p13.2
TAS2R30	12p13.2
TAS2R31	12p13.2
TAS2R38	7q34
TAS2R39	7q34
TAS2R40	7q34
TAS2R41	7q35
TAS2R42	12p13.2
TAS2R43	12p13.2
TAS2R45	12p13.2
TAS2R46	12p13.2
TAS2R50	12p13.2
TAS2R60	7q35

### 3.6 Sour taste receptors

Unlike sweet, bitter, and umami taste, GPCRs are not involved in the detection of sour taste. Instead, sour taste is caused by intracellular acidification (Lyall et al., 2001). The sour taste receptors mediating transduction have yet to be identified. Currently, it is shown that activation of the sour taste cell is induced by organic acids permeating the cell membrane, acidifying the cytoplasm resulting in the blockage of leak potassium (K^+^) channel KIR2.1 (Ye et al., 2016). In addition, protons entering the cell through a proton conductance channel further depolarize the sour taste cell (Ye et al., 2016). The proton conductance channel is located in a greater number at the top of the cell implying a function as ligand binding site (Richter et al., 2004); however, its molecular identity remains unknown.

Many candidate sour taste receptors have been proposed over the last three decades, such as the epithelial sodium channels, hyperpolarization‐activated cyclic nucleotide‐gated channels, acid‐sensing ion channels, and polycystic kidney disease protein‐like proteins (Huang et al., 2006). However, biophysical and mice gene studies have refuted these claims (Horio et al., 2011; Richter et al., 2004). New insights may be revealed after the recent discovery of a binary acid‐sensing mechanism in insects that may be evolutionarily conserved, and would involve mammalian homologs of the fly otopetrin family (Mi et al., 2021). Indeed, the protein Otopetrin‐1 (Otop1) is most promising as sour taste receptor (Tu et al., 2018). Otop1 emerged after selecting genes that encode proteins with multiple transmembrane domains combined with indicative expression patterns. Otop1 functions in vitro as sour (acid) taste receptor, that is, as a zinc‐sensitive proton conductance channel (Tu et al., 2018). The mechanism reported in *Drosophila melanogaster* involves gustatory receptor neurons selectively sensing low or high concentrations of acids. It is postulated that the acid‐taste signals antagonize each other in the brain, where the relative activity of low‐ and high‐acid determines the net behavioral response (Mi et al., 2021).

### 3.7 Salty taste receptors

Epithelial sodium channels (ENaCs) are thought to be the receptors responsible for detecting salt tastants, namely sodium chloride (Oka et al., 2013). Chandrashekar et al. (2010) showed ENaC‐associated Gα‐KO mice losing neural and behavioral responses to NaCl, implicating the ENaC‐associated Gα in the transduction of salty taste, and implying ENaC as receptor for salty tastants. Direct implicatory studies on ENaCs have yet to be successful (Bigiani, 2020). Kretz et al. (1999) provided evidence suggesting the existence of two distinct salty taste receptor cells, an amiloride‐sensitive taste receptor cell responsive to low concentrations of NaCl, and an amiloride‐insensitive taste receptor cell responding aversively to high concentrations of NaCl. However, associated nerve fibers showed similar sensitivity towards amiloride regardless of their NaCl sensitivity (Wu et al., 2015), indicating that salty taste transduction does not occur via separate nerve fibers. Instead, high concentrations of salt activate the bitter and sour taste receptor cells which are innervated by neural pathways associated with aversion. In mice, amiloride curbs NaCl response peaks.

Type III taste cells likely contain the salty taste receptors mediating high concentrations of salt (Wilson et al., 2019), whereas amiloride‐sensitive salt taste is suggested to be mediated by non‐type II/III cells, though the exact cell type remains ambiguous (Larson et al., 2020).

### 3.8 Expression of taste receptors

The TAS1R and TAS2R receptor genes are expressed in the human mouth, but also in extraoral tissues, including the nasal epithelium (Finger et al., 2003), brain (Chen et al., 2011), digestive system (Treesukosol et al., 2011) and other sites. Whereas the taste receptors in the oral cavity mediate the reception of taste, the receptor cells in extraoral tissue mediate processes such as nutrient detection, motor response induction and the maintenance of hormonal balances (Finger & Kinnamon, 2011). The gut can detect contaminants, induce gastric emptying and condition future aversion. Wauson et al. (2013) suggested gut‐expressed TAS1R1+TAS1R3 to regulate amino acid activation of transcription factors involved in gut health. Indeed, taste receptors in the gut initiate protective responses by detecting and responding to bacteria and bacterial signaling molecules (Turner et al., 2019).

Taste receptors behave differently per species. For example, unlike rodent receptor Tas1r1+Tas1r3, the human receptor TAS1R1+TAS1R3 is substantially more sensitive to L‐glutamate and L‐aspartate than to other L‐amino acids (Chaudhari et al., 2009). In addition to this higher sensitivity, humans can also taste various sweet substances such as monellin, thaumatin, aspartame, and neohesperidin dihydrochalcone (NHDC) that rodents cannot (Danilova et al., 1998). Zhao et al. (2003) engineered Tas1r2‐KO mice to express the human TAS1R2 gene, enabling them to respond to compounds that are normally tasteless to mice, including monellin, thaumatin and aspartame. Introduction of human TAS1R3 restored even the sweet taste of NHDC, suggesting TAS1R3 plays a key role in the binding of NHDC, but not of various other sweet substances, to the sweet taste receptor.

### 3.9 Genetic variations influencing taste

Gene polymorphisms in humans, such as Single Nucleotide Polymorphisms (SNPs), result in varying perceptions of taste, which can manifest itself in nutrient intake differences across geographic regions (Hwang et al., 2019). Although many genotype‐phenotype interactions are still poorly understood, genome wide association studies have been useful in identifying taste receptor candidate genes (Kim et al., 2003). Gene polymorphisms may influence sweet taste disproportionally more than umami taste, as the sweet taste receptor TAS1R2 knows far more genetic variations compared to the umami receptor counterpart TAS1R1 or the common component TAS1R3 (Kim et al., 2006). Additionally, polymorphisms in TAS1R3 affect the binding affinity to sweeteners (Nie et al., 2005), but not to amino acids (Nelson et al., 2002). A recent study provided proof that several SNPs are associated with interindividual differences in sensitivity and preference for umami (Chamoun et al., 2021). Farinella et al. (2021) showed that certain SNPs for TAS1R1 and TAS1R2 did associate with birth weight, whereas those selected for TAS1R3 did not.

## 4 TRANSDUCTION OF TASTE

### 4.1 Transduction of sweet, bitter, and umami taste

**FIGURE 4:**
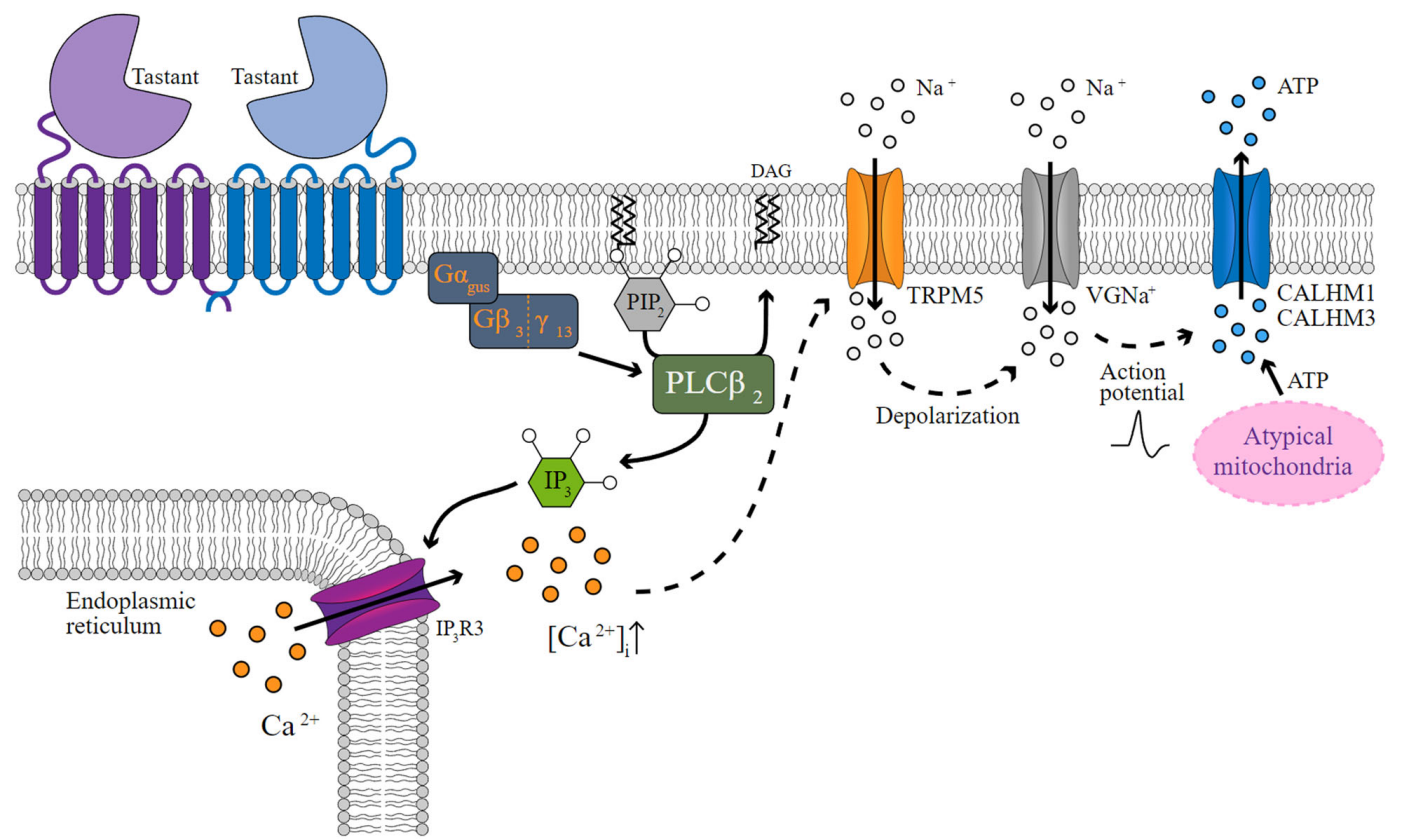
Taste transduction pathways for sweet, umami, and bitter taste. Tastant binding enables the GPCR dimer (top left) to cause GTP‐driven dissociation of gustducin subunit Gα‐ from subunits Gβ_3_/Gγ_13_. This triggers phospholipase Cβ_2_ (PLCβ_2_) to hydrolyze phosphatidylinositol 4,5‐bisphosphate (PIP_2_) into the second messenger inositol 1,4,5‐triphosphate (IP_3_) and diacylglycerol (DAG). The release of Ca^2+^ from the endoplasmic reticulum via the type 3 IP_3_ receptor (IP_3_R3) raises cytosolic calcium [Ca^2+^]_i_ and targets the channel protein TRPM5. Na^+^ inflow through voltage‐gated sodium channels (VGNa^+^) results in cell depolarization and the action potential triggers the opening of calcium homeostasis modulators 1 and 3 (CALHM1 and 3), through which adenosine triphosphate (ATP), produced by atypical mitochondria, is released onto gustatory afferent nerve fibers. Drawing inspired by Luddi et al. (2019)

Taste transduction begins when a taste compound, i.e., a ligand, enters the mouth and binds to the structurally compatible taste receptor in the taste pore. Ligand binding causes the GPCR to undergo a conformational change which enables it to function as a guanine nucleotide exchange factor (Denis et al., 2012). The resulting dissociation of subunit Gα‐gustducin from the dimeric subunit Gβ_3_/Gγ_13_ then signals downstream effectors (Figure 4). In this cascade, phospholipase C isoform β2 hydrolyses the membrane lipid phosphatidylinositol 4,5‐bisphosphate into diacylglycerol and the second messenger inositol 1,4,5‐triphosphate (IP3) (Liman et al., 2014). The release of IP3 within the taste cell induces a release of calcium (Ca^2+^) from the endoplasmic reticulum via the type 3 IP3 receptor. The raised levels of cytosolic calcium target TRPM5 (transient receptor potential cation channel subfamily M member 5) and cause an inflow of sodium through voltage‐gated sodium channels and subsequent cell depolarization (Liman et al., 2014). This action potential triggers the opening of calcium homeostasis modulators 1 and 3 through which adenosine triphosphate is released as neurotransmitter onto gustatory afferent nerve fibers (Romanov et al., 2018).

### 4.2 Transduction of sour taste

Sour taste transduction has yet to be elucidated as the sour taste receptor remains unidentified (Richter et al., 2004; Ye et al., 2016). Organic acids that permeate the cell membrane cause depolarization, acidify the cytoplasm and block the potassium (K^+^) channel KIR2.1 (Chang et al., 2010). In addition, protons entering the cell through a proton conductance channel further depolarize the cell (Ye et al., 2016). Cell depolarization results in an action‐potential, causing activation of neurons on the gustatory afferent nerve fibers.

### 4.3 Transduction of salty taste

Epithelial sodium channels (ENaCs) are thought to be the receptors responsible for detecting salty taste (Chandrashekar et al., 2010). The aversive response from high concentrations of salt (>300 mM NaCl or KCl) is mediated by the two aversive nerve fibers associated with the bitter and sour taste (Oka et al., 2013).

Oka et al. (2013) postulated that some TAS2R‐receptors may bind to high concentrations of salt, or that a signaling component such as an ion channel activates a bitter taste cell. Additionally, high ionic strength inhibits the carbonic acid receptor, carbonic anhydrase 4 (Car4), which may lead to an increase of intracellular protons and trigger the associated aversive neural pathway (Chandrashekar et al., 2009). See Table 3 for an overview of the taste‐associated receptors, their ligands, and implicated transduction pathways.

**TABLE 3:** Overview of taste‐associated receptors, their ligands, and related transduction pathways

**Taste quality**	**Taste receptors**	**Ligands**	**Examples**	**Transduction pathways**
Sweet	TAS1R2+TAS1R3	Sugars, sugar substitutes, sweet amino acids, peptides, proteins	Sucrose, glycine, aspartame, monellin	PLCB2
Sweet	TAS1R3+TAS1R3	Low affinity sweet taste receptor, does not bind to sugar subsitutes or d‐amino acids	Sucrose	PLCB2
Umami	TAS1R1+TAS1R3	Mostly l‐amino acids	Alanine, serine, glutamine	PLCB2
Umami	GRM1−4	Glutamate, some analogs	Glutamate, L‐AP4	Unknown
Bitter	∼25 TAS2 receptors	Different for each receptor	Caffeine, quinine, PROP, PCT	PLCB2
Sour	Otopretin‐1	Mainly organic acids	Acetic acid, citric acid	KIR2.1
Salt	ENACs	Salts	NaCl	Ion channel; Car4

GRM1−4, GRM1, GRM2, GRM3, GRM4; PLCβ2, phospholipase C isoform β2; L‐AP4, L‐(+)−2‐amino‐4‐phosphonobutyrate; PROP, 6‐n‐propylthiouracil; PCT, phenylthiocarbamate; KIR2.1, potassium (K+) channel; ENaC, epithelial sodium channel; Car4, carbonic anhydrase 4.

### 4.4 Nerve complex

The neural response is mediated by the cranial nerve, which emerges directly from the brain and brainstem. Three nerves mediate the sense of taste, the chorda tympani nerve (cranial nerve VII), the glossopharyngeal nerve (cranial nerve IX), and the vagus nerve (cranial nerve X). These nerves enter the brainstem at the pontomedullary junction where the signals travel toward the end of the stem of the medulla oblongata, synapsing at the nucleus solitary. The signals are transmitted through the thalamus to the gustatory cortex and the hypothalamus. Finally, at the orbitofrontal cortex, the gustatory (sense of taste), olfactory (sense of smell) and somatosensory (sense of mouth feel, e.g., texture,) signals are unified, underlying the sense of flavor (van der Werf et al., 2017).

### 4.5 Neural coding of taste receptors

Neural coding studies the processing and transformation of information by neurons (Richmond, 2009). Two different models for encoding taste information have been proposed. The labeled line model articulates that each taste receptor cell is innervated by individually tuned, nonoverlapping nerve fibers (Wu et al., 2021). This means that taste is perceived by the brain based on which nerve fiber is sending the signal. The across‐fiber model on the other hand postulates that taste signals are transmitted by broadlytuned, overlapping nerve fibers (Wu et al., 2021). This would mean that any incoming signal may transmit any of the five taste qualities. The difference between the two models may be illustrated if the brain were imagined to be a traffic camera overlooking a highway. With the labeled line model, it would detect in which lane the traffic occurs whereas with the across‐fiber model, it would detect the pattern (order and timing) of the traffic, regardless of the traffic lane.

The across‐fiber model has long been the predominant model (Frank & Pfaffmann, 1969; Ohla et al., 2019), but since the early 2000s, major advancements were made in molecular biological, electrophysiological, genetical, and behavioral studies, which provided evidence for the labeled line model (Mueller et al., 2005). Zhao et al. (2003) genetically engineered mice to express a modified opioid receptor in the sweet taste cells; these mice became selectively attracted to the normally tasteless opioid spiradoline. Mueller et al. (2005) did similar studies with a TAS2R bitter receptor expressed in sweet taste cells, yielding mice that were attracted to bitter compounds of the glucopyranoside family. The results indicated that the hosting taste cells dictate attractive and aversive behaviors, supporting the labeled lines model. However, later studies with calcium imaging and in vivo neuron studies in anesthetized mice provided evidence for the existence of a combination of both models (Wu et al., 2015). About half of the gustatory sensory ganglion neurons were stated to be tuned to a single taste quality, the other half being responsive to multiple tastants (Wu et al., 2015). Taste cells and neurons therefore respond selectively or broadly to taste stimuli; also temporal response patterns seem to be important for the development of taste behavior (Ohla et al., 2019).

## 5 THE CHEMISTRY OF UMAMI TASTANTS

### 5.1 Taste of amino acids

The taste of amino acids is dependent on the hydrophobicity, size, charge, isoelectric point, chirality of the alpha carbon, and the functional groups on the side chain of the amino acid. Most l‐amino acids are perceived as sweet or bitter, whereas many d‐amino acids elicit a sweet taste (Kawai et al., 2012; Solms et al., 1965). Additionally, the l‐amino acids glycine, alanine, serine, glutamic acid, asparagine and glutamine have been shown to elicit umami taste when tested at high concentrations (Kawai et al., 2012). The predominant umami tastant is l‐glutamate, widely used as seasoning in the form of the sodium salt of glutamic acid, monosodium l‐glutamate (MSG). The d‐isomer of MSG does not elicit umami taste (Komata, 1990). Interestingly, a recent study found that both isomeric forms (l‐ and d‐) of monosodium pyroglutamate were major umami compounds in potato chips (Zhang & Peterson, 2018).

### 5.2 Structure–Function relationship of glutamic acid derivatives

**FIGURE 5:**
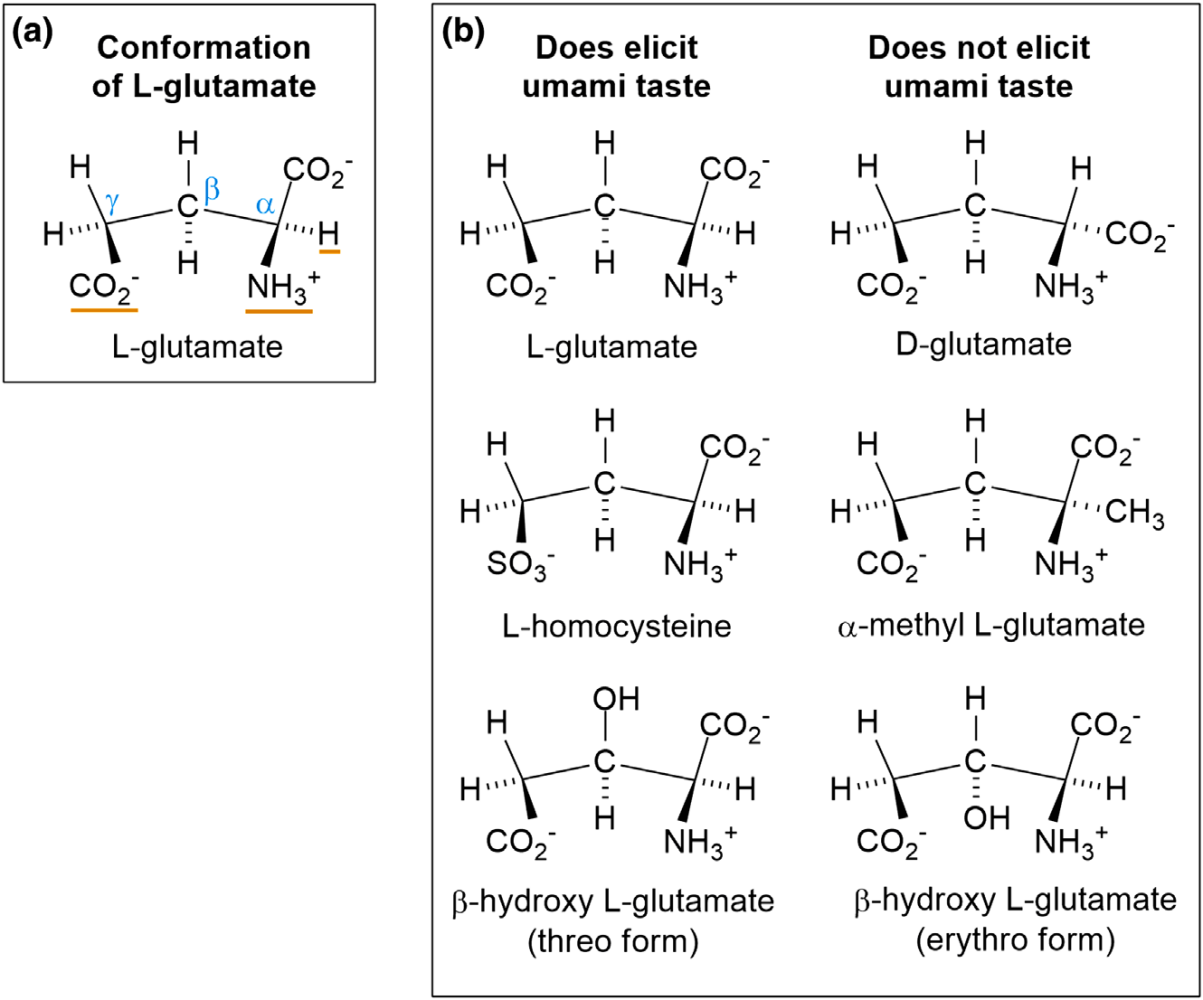
l‐Glutamate and the relationship between umami taste and the chemical structures of related compounds. A. The linear molecule of glutamate forms an eclipsed conformation promoted by electrostatic interaction of CO_2_^–^ and NH_3_^+^ which allows structural attachment to the umami taste receptor, facilitated by the three radicals of the α‐amino, γ‐carboxyl, and α‐hydrogen atoms (underlined).B. The three structures on the left elicit umami taste, whereas the three related structures on the right do not (based on Komata, 1990)

As established, the chemical structure of tastants affects the corresponding taste. In particular, variations of glutamate are known to elicit umami, that is, l‐homocysteate and β‐hydroxy‐l‐glutamate in threo form (Figure 5B).

The acetylation, esterification, or methylation of MSG results in loss of umami taste (Komata, 1990). Even simple isomers of umami tastants fail to elicit umami taste, indicating the delicate relationship between chemical structure and biological effect. In the replacement of MSG's beta‐hydrogen by a hydroxy radical, its threo form produces umami taste but its erythro form does not (Figure 5B; Komata, 1990). Both monosodium l‐aspartate and monosodium l‐amino pimelate produce weaker umami tastes compared to MSG (Komata, 1990) as they have one less and two extra carbon atom(s), respectively. By contrast, monosodium l‐homocystate, formed through the replacement of MSG's gamma‐carboxyl radical by a sulfonium radical, produces a stronger umami taste than MSG (Komata, 1990). 

For a compound to elicit umami taste, it must be able to structurally attach to the umami taste receptors. The underlying mechanism may have been adequately explained by Kaneko (1963). According to his model, “it is necessary for the realization of umami taste that an α‐amino radical electrically pulls a γ‐carboxyl radical to form a penta carbon ring so that the ring can have a flat configuration built with the three radicals of α‐amino, γ‐carboxyl and α‐hydrogen atom” (Figure 5A). Both monosodium d‐glutamate and monosodium α‐methyl l‐glutamic acid cannot build such a flat configuration; therefore, attachment to the umami taste receptor is impossible. Moreover, the reduced umami taste intensity of MSG under highly acidic or highly alkaline conditions might be explained by the way the MSG's γ‐carboxyl radical is in the state of –COOH in acidic conditions, while in alkaline conditions, the MSG's α‐amino radical is in the state of –NH_2_. In such conditions, the static electrical strength binding the α‐amino radical with the γ‐carboxyl radical becomes low (Komata, 1990).

### 5.3 Umami tastant synergism

**FIGURE 6:**
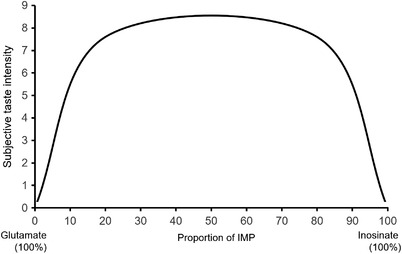
Umami taste synergism. Synergism of glutamate and inosine monophosphate (IMP) yields an inverted U shape when taste intensity (*y*‐axis) is plotted against compound proportion (*x*‐axis). Based on data from Yamaguchi et al. (1967)

In humans, nucleotides have been demonstrated to show synergism with umami tastant l‐glutamate (Wu et al., 2021). First reported in the 1960s, guanosine monophosphate (GMP) and inosine monophosphate (IMP) were demonstrated to strongly enhance umami taste in the presence of glutamate (Figure 6; Yamaguchi, 1967). Various other nucleotides also demonstrate this synergism to varying degrees (Yamaguchi & Ninomiya, 1998). For millennia, cuisines all across the world have been combining glutamate‐containing foods with nucleotide‐containing foods. Prominently seen in stocks and broths such as the combination of kombu seaweed with dried bonito in Japan, or the combination of beef with onion, carrot, and celery in the West, respectively (Kurihara, 2015; Yamaguchi & Ninomiya, 2000). Yamaguchi (1967) investigated the synergism by measuring the umami taste intensity of a mixture containing MSG and IMP (0.05 g/dl). Mixtures with varying concentrations of IMP (0−100%) were tested. Umami taste perception strongly increased when the proportion of IMP was between 20% and 80% (Figure 6).

Not all nucleotides, only 5′‐ribonucleotides, produce a synergistic effect with glutamate (Komata, 1990). Three conditions are required for nucleotides to be able to structurally bind to the VFTD and produce the synergism with glutamate. Firstly, the base moiety must be a purine. Secondly, the 6′‐carbon in the purine ring must be a hydroxyl radical. Thirdly, the ribose moiety must be phosphorylated at the 5′‐carbon. When the ribose moiety is phosphorylated on the 2′‐ or 3′‐carbon, umami taste is lost. The number of phosphate groups on the 5′‐carbon does not affect the synergistic response.

## 6 MOLECULAR INTERACTIONS THAT ELICIT UMAMI TASTE

### 6.1 Ligand binding site

As introduced earlier, the amino acid‐terminal ectodomain (ATD) of the umami taste receptor contains a binding site known as the venus flytrap domain (VFTD), which consists of two lobes, an upper and lower lobe (Figure 3) (Pin et al., 2003). The two lobes can take on an open or closed conformation; ligand binding results in the stabilization of the closed conformation where the ligand essentially folds both protein lobes (Muto et al., 2007). Nucleotides such as GMP and IMP bind to the outer section of the VTFD and further stabilize the closed conformation (Mouritsen & Khandelia, 2012; Zhang et al., 2008). Unlike the principal umami taste receptor TAS1R1+TAS1R3, GRM1 nor GRM4 seem to display the described synergism (Kurihara, 2015).

### 6.2 Molecular mechanism

The molecular mechanism underlying the umami taste, and, by extension the umami synergy, was first elucidated by Zhang et al. (2008) using site‐directed mutagenesis and molecular modeling. TAS1R1 taste receptor variants were generated to determine which residues are involved in l‐glutamate and IMP binding. The residues S172, D192, Y220, and E301 each are essential to mediate l‐glutamate binding and the residues H71, R277, S306, and H308 each for IMP binding (Zhang et al., 2008). Dang et al. (2019) and Liu et al. (2019) reported various other key residues essential for ligand binding and thus necessary for umami taste transduction (Table 4).

**TABLE 4:** Key residues involved in binding of umami compounds to the umami taste receptors TAS1R1 and TAS1R3

**Ligand**	**Lobe**	**Key residues**	**Hydrogen bond interaction**	**Ref**.
MSG	TAS1R1	D147, A170, D192, Y220, E301, A302	α‐Amino acid moiety	*1*
MSG	TAS1R1	T149, S172, R277,	Carboxylate moiety	*1*
IMP	TAS1R1	S172, T149	Nitrogenous base	*1*
IMP	TAS1R1	S48, N69, S276, R277	Phosphate group	*1*
IMP	TAS1R1	H71, S306, H308	Not described	*2*
Peptides	TAS1R3	Y131, Q326, G328, H388, E453	–	*3* *
Peptides	TAS1R3	Y143, S147, S170, R327, D190	Carboxylate moiety 1	*4* *
WSA	TAS1R1	T149, S172, R277		*2*
WSA	TAS1R3	S300, A302	Carboxylate moiety 2	*2*
BMP	TAS1R1	N69, Y220, S276, R277, A302, S385	D3, E4, E5	*2*
BMP	TAS1R3	R151	C‐terminal A8	*2*

Abbreviations: BMP: beefy meaty peptide; IMP: inosine monophosphate; MSG: monosodium glutamate; WSA: sodium succinate.

* Numbering according to protein database entry NP_689414 (www.ncbi.org).

(1) Zhang et al. (2008) & Liu et al. (2019), (2) Liu et al. (2019), (3) Dang et al. (2019), (4) Zhang et al. (2021).

While the binding mechanism was unknown, Polanski and Gieleciak (2003) postulated that at the receptor cavity, a series of interacting regions are established which produce residue‐binding sites. Then, the rotatable chemical bonds within the ligand and the residue acceptor are both optimized and molecular binding proceeds (Polanski & Gieleciak, 2003). Van der Waal's‐, electrostatic‐, π–π interactions, and hydrogen bonds mediate ligand binding to the receptor. Despite low sequence identity of TAS1R1 and TAS1R3 with GRM proteins, residues near the hinge of the VFT domain that connect the two lobes are conserved. Zhang et al. (2008) therefore proposed that the glutamate binding position in TAS1R1 follows that in the GRM proteins.

Dang et al. (2019) simulated the umami taste receptor TAS1R1+TAS1R3 and found that MSG bound to TAS1R1 nearly doubled the size of the binding cavity of TAS1R3 (534.125 A^3^ to 1135.73 A^3^). This increased cavity size allows di‐ and tripeptides to bind with TAS1R3. Long peptides with high molecular weights could not bind when MSG was already bound (Dang et al., 2019).

### 6.3 Taste of peptides

Di‐ and tripeptides generally show weaker umami taste compared to MSG; however, at 0.8 mM concentrations, the umami taste of Gly–Glu and Ala–Glu–Ala was stronger than that of MSG (Ohyama et al., 1988; Soldo et al., 2003). Synthetic hexa‐ and heptapeptides showed weaker umami taste than naturally formed hexa‐ and heptapeptides (Zhang et al., 2017). Small oligopeptides called “flavor” peptides also elicit umami taste (Dang et al., 2015; Su et al., 2012). Some natural octapeptides elicit umami taste while synthesized octapeptides sometimes not only elicit umami taste, but also sweet and sour tastes (Zhang et al., 2017). A recent study by Zhang et al. (2021) reported the identification of 14 oligopeptides isolated from a saltwater clam, all of which showed umami and umami‐enhancing effects (Table 4).

#### 6.3.1 Peptide interactions with cations

Salts, such as sodium chloride (NaCl) enhance the flavor response of other compounds, including amino acids. Only low amounts of NaCl are needed to increase the umami perception (Kurihara, 2015; Ugawa et al., 1992). The presence of salts does not affect amino acid affinity to the taste receptors since the umami taste threshold remains unaltered regardless of salt presence (Ugawa et al., 1992). The solvability of different salts may influence the taste perception; where a quick‐to‐dissolve salt would have a higher taste intensity but a low temporal stay, vice versa for salts with a lower solvability. Also the umami synergism between MSG and nucleotides is stimulated by NaCl (Ugawa et al., 1992).

Nakata et al. (1995) examined the interactions of acidic peptides with Na^+^ and K^+^ cations. As pH levels were raised to pH 6 using either NaOH or KOH solutions, the sodium salts of the acidic dipeptides showed both umami and salty tastes. In contrast, the potassium salt peptides elicited an indistinct taste that could not be categorized as umami or salty (supplementary). Of course, peptide sequence is key in eliciting umami taste (Yu et al., 2017). Peptides with side groups adjusted to their corresponding binding region, that is, having appropriate sizes and charges, will likely enhance umami taste (Yu et al., 2017).

#### 6.3.2 Formation of umami compounds

The principal umami tastant l‐glutamate is formed during fermentation processes through proteolysis or converted from glutamine by glutaminase activity of lactobacilli (Zhao et al., 2016). Additionally, two other umami compounds are also formed: pyroglutamic acid (pGlu) and pGlu‐Pro‐X peptides. Pyroglutamyl di‐peptides are formed from α‐glutamyl‐ or α‐glutaminyl dipeptides during heating by cyclization, and from pyroglutamic acid and free amino acids by pGlu cyclase (Zhao et al., 2016). Remarkably, subthreshold concentrations of various pyroglutamyl peptides and Amadori products enhance the umami taste of soy sauce (Kaneko et al., 2011). These compounds are generally perceived as bitter at suprathreshold concentrations.

The taste of α‐Glu di‐ and tripeptides in cheese is strongly dependent on the hydrophobicity of the second amino acid (Arai et al., 1973). Hydrophilic amino acids second in chain such as Asp, Thr, Ser, and Glu elicit the umami taste, whereas moderately hydrophobic amino acids such as Gly, Ala, Pro, Val, Ile and the strongly hydrophobic Leu, Tyr, and Phe elicit flat and bitter tastes, respectively (Arai et al., 1973; Zhu et al., 2016). Hydrophilic oligopeptides containing Glu also elicit umami taste, particularly when Glu is attached at the N‐ or C‐terminal of the peptide (Kim et al., 2015). Through proteolysis of casein, α‐Glu‐X is formed, yet the corresponding taste is not connected to γ‐Glu‐X (Toelstede et al., 2009; Toelstede & Hofmann, 2009).

In dried meat, the intensity of the salt taste is correlated with the concentrations of Glu and Asp (Careri et al., 1993). In cheese, subthreshold levels of arginine increased the salt taste intensity (Toelstede et al., 2009; Toelstede & Hofmann, 2008). In soy sauce, subthreshold levels of bitter tasting amino acids increased the umami taste intensity (Lioe et al., 2004).

## 7 UMAMI AND HEALTHY AGING

Sodium levels in food may be partially reduced due to the enhancing effect of umami on overall taste, without sacrificing palatability (Morita et al., 2020). Currently, global daily sodium intake exceeds the recommendation of the World Health Organization (Thout et al., 2019). Excessive consumption of salt can have adverse effects, including high blood pressure, heart disease, and stroke. Studies show a possible sodium reduction of up to 40% when adding glutamate, depending on the sources and types of food, and the presence of nucleotides (Rosa et al., 2021; Tanaka et al., 2021).

Few studies exist on umami potentially reducing consumption of fats. Bellisle (2008) reported MSG rescuing some palatability in two of three dishes containing 30% less fat, while maintaining an overall decreased energy intake. Imada et al. (2014) showed that adding MSG to a “preload” broth decreased subsequent fat intake. This was further supported by a study from Miyaki et al. (2015), which reported a decreased consumption of high‐fat foods in overweight and obese women by adding MSG to vegetable soup. In volunteers with low glutamate intake and low protein intake, the state of “glutamate undernutrition” suppressed T1R3 gene expression and this improved by adding MSG (Beppu et al., 2021). In these subjects also high salt and sugar intake was corrected by supplementation with MSG.

Umami may increase nutritional intake in the elderly (Sasano et al., 2015). In the elderly, hyposalivation is common due to impaired taste‐ and smell receptors, caused by aging and possible side effects from medication (Diep et al., 2021). Symptoms of hyposalivation may include dry mouth, discomfort, inadequate nutrition resulting from loss of taste, pain, and a decline in dental health (Golež et al., 2021). Umami is shown to stimulate long‐lasting saliva secretion, appetite during consumption, postingestive satiety, and overall improved health and weight (Masic & Yeomans, 2014; Uneyama et al., 2009). These benefits are key in the prevention of undernourishment in the elderly since malnutrition decreases health prospects and quality of life.

## 8 CONCLUDING REMARKS

Understanding taste is key for optimizing the palatability of seaweeds and other non‐animal‐based foods rich in protein. A better understanding of the interactions between taste‐active compounds, and the contribution of “subthreshold compounds” to the overall taste, will improve the quality of food products with regard to taste, intake, and food safety. Umami ingredients may help to reduce the consumption of salts and fats in the general population and increase food consumption in the elderly.

## AUTHOR CONTRIBUTION

Johan Diepeveen: Conceptualization; visualization; writing – original draft. Tanja C. W. Moerdijk‐Poortvliet: Conceptualization; funding acquisition; supervision; writing – review & editing. Feike R. van der Leij: Conceptualization, Funding acquisition, Supervision, Visualization, Writing – review & editing

## CONFLICT OF INTEREST

The authors declare that there is no conflict of interest.

## Supporting information

Table 5/Supplemental Table 1. Taste of free amino acids
Table 6/Supplemental Table 2. Peptides reported to have umami taste
