# Supplementary material for: Molecular insights into human taste perception and umami tastants: A review
Source: J Food Sci. 2022 Mar 17;87(4):1449–65. doi: 10.1111/1750-3841.16101 (PMC9314127; doi:10.1111/1750-3841.16101)
Supplement: Supplementary file 1 — Table 5/Supplemental Table 1. Taste of free amino acids Table 6/Supplemental Table 2. Peptides reported to have umami taste [file JFDS-87-1449-s001.docx]

Appendices

Table 5 / Supplemental Table 1. Taste of free amino acids.

	Taste reported by					
	**Solms (1969)**	**Wieser et al. (1977)**	**Kawai et al. (2012)**	**Solms (1969)**	**Wieser et al. (1977)**	**Kawai et al. (2012)**
**Amino acid**		**L-form**			**D-form**	
Gly	Sweet	Sweet	Sweet, umami	-	-	-
Ala	Sweet	Sweet	Sweet, umami	Flat	Sweet	Sweet,
Ser	Flat	Sweet	Sweet, umami, sour	Flat	Sweet	Sweet, sour
Thr	Flat	Sweet	Sweet, sour	Flat	Sweet	Sweet,
Tyr	Bitter	Bitter	-	Sweet	Sweet	-
Cys	Sulfurous	Sulfurous	Bitter, sweet	Sulfurous	Sulfurous	-
Met	Sulfurous, meaty, sweet	Sulfurous	Bitter, sweet	Sulfurous, meaty, sweet	Sweet, sulfurous	Sweet, bitter
Val	Flat	Bitter	Bitter, sweet	Flat	Sweet	Sweet, bitter
Leu	Bitter	Bitter	Bitter	Sweet	Sweet	Sweet, bitter
Ile	Flat	-	Bitter	Flat	-	-
Phe	Bitter	-	Bitter	Sweet	-	Sweet, bitter
Trp	Bitter	-	Bitter	Sweet	-	Sweet, bitter
Asp	Flat	Neutral	Sour	Flat	Sweet	Sour
Glu	Unique, "glutamate"	Sour, glutamate	Sour, umami	Flat	Sour, neutral	Sour
Asn	-	-	Sour, umami	-	-	Sweet, sour
Gln	-	Neutral	Sweet, umami	-	Sweet	Sweet, sour
His	Flat	Bitter	Bitter	Sweet	Sweet	Sweet, bitter
Lys	Flat	Sweet, bitter	Bitter	Flat	Sweet	-
Arg	Flat	Bitter	Bitter	Sweet	Neutral	Bitter, sweet
Pro	Flat, Sweet	Sweet, bitter	Sweet, bitter	Flat	Neutral	Bitter

Table 6 / Supplemental Table 2. Peptides reported to have umami taste

Type and number of peptides	Amino acid sequence of umami peptide	Source	Taste as reported (pH)	Reported threshold concentration of umami	Authors
25 Dipeptides	Asp-Ala	Soy sauce	Umami	-	Oka and Nagata (1974)
	Ala-Asp	Synthesized	Bitter > umami	13 mM	Ohyama et al. (1988)
	Ala-Glu	Synthesized	Umami (neutral)	1.5 mM	Ohyama et al. (1988)
	Asp-Asp	Synthesized	Salty/umami (6.0)	4.79 mM	Tamura et al. (1989)
	Asp-Glu	Synthesized	Salty/umami (6.0)	1.25 mM	Tamura et al. (1989)
	Asp-Leu	Synthesized	Umami (neutral)	2.5 mM	Ohyama et al. (1988)
	Fru-Glu	Soy sauce	Umami	0.8 mM	S. Yamamoto et al. (2014)
	Glu-Asp	Proteinase-modified soybean protein	Brothy	-	Arai et al. (1972)
		Synthesized	Brothy (6.0)	200 mg%	Arai et al. (1973)
		Fish protein hydrolysate	MSG-like	200 mg%	Noguchi et al. (1975)
		Synthesized	Salty/umami (6.0)	3.14 mM	Tamura et al. (1989)
		Fish protein hydrolysate	Umami	7.6 mM	Schindler et al. (2011)
	Glu-Glu	Proteinase-modified soybean protein	Brothy	-	Arai et al. (1972)
		Synthesized	Brothy (6.0)	1% solution	Arai et al. (1973)
		Fish protein hydrolysate	MSG-like (6.0)	150 mg%	Noguchi et al. (1975)

	Synthesized	Salty/umami (6.0)	2.73 mM	Tamura et al. (1989)
	Synthesized	Umami	1% (g/mL)	Maehashi et al. (1999)
	Fish protein hydrolysate	Umami	5.4 mM	Schindler et al. (2011)
Glu-Leu	Synthesized	Umami	3 mM	Ohyama et al. (1988)
Glu-Lys	Synthesized	Umami (6.0)	3.12 mM	Tamura et al. (1989)
Glu-Orn	Synthesized	Umami/sour	3.12 mM	Tamura et al. (1989)
Glu-Ser	Proteinase-modified soybean protein	Brothy	-	Arai et al. (1972)
	Synthesized	Weak brothy (6.0)	-	Arai et al. (1973)
	Fish protein hydrolysate	MSG-like (6.0)	200 mg%	Noguchi et al. (1975)
	Fish protein hydrolysate	Umami	8.5 mM	Schindler et al. (2011)
Glu-Thr	Synthesized	Brothy taste	-	Arai et al. (1973)
Glu-Val	Synthesized	Umami/sweet	1% (g/mL)	Maehashi et al. (1999)
Gly-Asp	Synthesized	Umami	6 mM	Ohyama et al. (1988)
Gly-Glu	Synthesized	Umami > bitter	0.8 mM	Ohyama et al. (1988)
Leu-Glu	Synthesized	Umami > bitter	1.5 mM	Ohyama et al. (1988)
Lys-Gly·HCl	Synthesized	Salty / umami (6.0)	1.22 mM	Tamura et al. (1989)
Orn-Orn·2HCl	Synthesized	Umami	1.5 mM	Tamura et al. (1989)
Orn-Ala·HCl	Synthesized	Salty/Umami	1.25 mM	Tamura et al. (1989)

pGlu-Pro	Deamidated wheat gluten hydrolysate	MSG-like	-	Schlichtherle-Cerny and Amado (2002)
Thr-Glu	Fish protein hydrolysate	MSG-like	300 mg%	Noguchi et al. (1975)
	Fish protein hydrolysate	Umami	12.1 mM	Schindler et al. (2011)
Val-Asp	Synthesized	Bitter>umami	25 mM	Ohyama et al. (1988)
Val-Glu	Synthesized	Umami> Bitter	1.5 mM	Ohyama et al. (1988)
